# Information Entropy-Guided Multi-Scale Feature Fusion for Crowd Density Estimation

**DOI:** 10.3390/e28060617

**Published:** 2026-05-30

**Authors:** Zixun Liu, Tianle Yang, Yongjie Wang

**Affiliations:** 1 School of Mechanical Engineering, Shijiazhuang Tiedao University, Shijiazhuang 050043, China; 2 Tianjin Fire Research Institute of Emergency Management Ministry, Tianjin 300381, China

**Keywords:** information entropy, density-aware attention, multi-scale feature fusion, crowd counting, transformer

## Abstract

The spatial heterogeneity of crowd distributions poses significant challenges for density estimation. Dense regions exhibit high local information entropy due to severe occlusion and feature ambiguity, while sparse regions and backgrounds carry progressively lower informational complexity. To address this, we propose an entropy-inspired crowd density estimation framework that allocates computational attention in proportion to the local information complexity of crowd regions. A Density-Guided Map (DGMap), constructed from nearest-neighbor distance statistics of head annotations, serves as a proxy for local information entropy, enabling the model to differentiate among dense, sparse, and isolated pedestrian regions. The proposed network, termed DGCC-Net, comprises four components: a Twins-Transformer backbone for hierarchical feature extraction, a Local Attention Module (LAM) that enhances high-resolution features through multi-scale receptive fields and rotational attention, a Multi-Level Feature Fusion Module (MLFM) with cross-scale dense connectivity and learnable branch weights for integrating semantic and spatial information, and a Density Guidance Module (DGM) supervised by the entropy-inspired DGMap to achieve density-adaptive feature refinement. Extensive experiments on four benchmark datasets (ShanghaiTech PartA, UCF-QNRF, UCF_CC_50, and JHU-Crowd++) demonstrate that DGCC-Net achieves competitive or state-of-the-art performance, validating the effectiveness of entropy-inspired attention allocation in heterogeneous crowd scenarios.

## 1. Introduction

**Figure 1:**
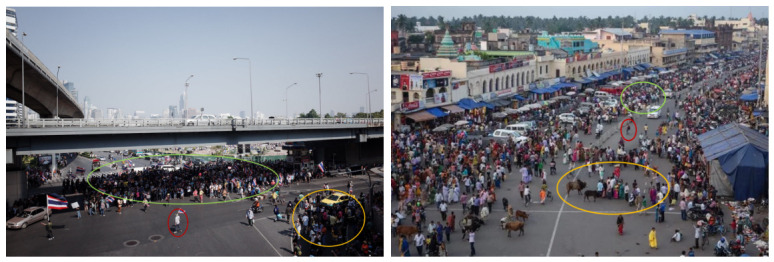
The red circle represents isolated pedestrians, the orange circle represents sparse crowds and the green circle represents dense crowds.

In intelligent video surveillance systems, crowd density estimation achieves accurate crowd size prediction by analyzing real-time images from monitoring devices. This technology provides significant value in applications, such as crowd behavior analysis and danger prediction. In particular, it plays an indispensable role in preventing stampedes and controlling potentially hazardous crowd gatherings. In real-world surveillance scenarios, unpredictable crowd activity leads to random variations in location and density, resulting in diverse distribution patterns. As shown in Figure 1, these patterns include dense crowds, sparse crowds, and isolated pedestrians.

From an information-theoretic perspective, these diverse distribution patterns correspond to a spatial variation in local information complexity: dense regions exhibit high feature ambiguity due to inter-occlusion, sparse regions exhibit moderate complexity with clearer boundaries, and background areas carry minimal task-relevant information. This complexity gradient suggests that an effective counting model should adaptively allocate its representational capacity according to local density characteristics.

To address this challenge, many studies have proposed various methods. Rong et al. [1] utilized coarse and fine-grained attention maps to identify crowd regions with varying densities. Liu et al. [2] added an auxiliary segmentation branch for dense crowd regions. Pan et al. [3] utilized attention mechanisms to differentiate between dense and sparse regions, constructing a dual-branch counting network. Xie et al. [4] proposed a multi-scale super-resolution-guided network to address crowd occlusion. Gao et al. [5] introduced the Swin Transformer [6] for deep feature extraction combined with feature pyramids. Yi et al. [7] employed deconvolution modules to restore high-resolution information for dense scenarios.

Despite this progress, existing methods lack an explicit information-theoretic formulation for density-aware feature allocation. While binary segmentation masks and coarse attention maps provide implicit density awareness, they do not capture the continuous entropy gradient inherent in crowd distributions. To our knowledge, no prior work has systematically employed information entropy principles to guide feature allocation across multiple density levels.

Inspired by these observations, this paper proposes a Density-Guided Crowd Counting Network (DGCC-Net) grounded in the principle of entropy-inspired proportional attention allocation. The model uses a Density-Guided Map (DGMap) based on nearest-neighbor distances to assist the model in learning various crowd distribution patterns. DGCC-Net comprises four main components: a Twins-Transformer feature extraction network, a Local Attention Module (LAM), a Multi-Level Feature Fusion Module (MLFM), and a Density Guidance Module (DGM). The model is evaluated on four datasets—ShanghaiTech PartA, UCF-QNRF, UCF_CC_50, and JHU-Crowd++—achieving competitive counting results across low-density, medium-density, and high-density scenarios. The main contributions are as follows:

1. We propose a practical density-level guidance mechanism inspired by information entropy principles. By constructing continuous density-level maps from nearest-neighbor distance statistics—serving as a density-based spatial entropy proxy—the mechanism captures the density gradient across dense, sparse, and isolated pedestrian regions. Unlike binary segmentation masks used in prior work, this multi-level characterization provides finer-grained attention guidance, enabling the model to adaptively allocate computational resources according to local feature complexity. While individual components (Transformer backbone, attention module, multi-scale fusion) draw from established techniques, the principal contribution lies in the unified density-level guidance framework that provides a principled criterion for capacity allocation across heterogeneous regions. We support this mechanism with both a formal mathematical analysis linking nearest-neighbor distance to local information entropy and quantitative empirical validation.
2. We integrate multiple complementary modules into the network, including a Local Attention Module (LAM) with multi-scale receptive fields and rotational attention, and a Multi-Level Feature Fusion Module (MLFM) with cross-scale dense connectivity and learnable branch weights, to improve counting accuracy across heterogeneous density scenarios.
3. We conduct comprehensive experiments, including ablation studies on multiple datasets, fine-grained sub-module analysis, hyperparameter sensitivity analysis, computational complexity comparison, and cross-dataset generalization tests, empirically validating that entropy-inspired attention allocation consistently improves counting accuracy.

The structure of this paper is as follows. First, the overall architecture and key components of DGCC-Net are introduced. Then, the specific settings for model training and the loss functions are described. Next, the experimental results comparing the model’s performance on different datasets are presented, along with an analysis of the effectiveness of each module. Finally, the last section summarizes the research findings and contributions.

## 2. Related Work

Over the past few years, there has been a gradual shift from traditional methods [8,9] to CNN-based methods [10,11,12] to solve crowd counting problems. In this section, we will primarily discuss two frequently used CNN-based methods for counting.

### 2.1. Multi-Scale Information Fusion Methods

Qiu et al. [13] proposed a multi-scale feature encoder constructed with dilated convolutions of varying expansion rates. By stacking this module, they adopted a coarse-to-fine strategy to generate high-quality density maps. Additionally, a multi-scale structural similarity loss was introduced to guide the network in learning local correlations within density maps. Building on the principle that scale is inversely proportional to object depth, Zhao et al. [14] incorporated a depth-aware embedding module into their network architecture, leveraging depth information to spatially recalibrate raw features and generate scale-sensitive representations. Qian et al. [15] developed a multi-scale network with a U-shaped structure, harnessing the global receptive fields of Twins Transformers [16] hierarchical U-Net layers to extract and aggregate semantic and spatial features across multiple levels. Extending these advancements, Yi et al. [7] proposed a counting model with a feature pyramid structure, aligning branch features to diverse scales using receptive field-specific convolutions and adjusting sensitivity to perspective variations through learnable perspective-aware parameters.

### 2.2. Attention Guided Methods

Miao et al. [17] reduced background interference in counting performance by generating attention masks from low-level features. Rong et al. [1] employed a coarse-to-fine attention mechanism to better integrate features across hierarchical levels while focusing on crowd regions. Wang et al. [18] proposed a segmentation-guided attention network, introducing explicit attention maps generated from point annotations as additional supervision to enhance visual feature extraction for density map estimation.

### 2.3. Information-Theoretic Methods in Visual Feature Learning

Information theory has provided foundational tools for understanding and improving deep learning systems. The information bottleneck principle [19] formalizes the trade-off between compression and prediction in neural networks, inspiring architectures that retain task-relevant information while discarding noise. In the context of image analysis, entropy-based measures have been employed for feature selection [20], active learning [21], and attention allocation [22]. Shannon entropy and its variants serve as natural indicators of local image complexity, with high-entropy regions typically demanding more computational resources for accurate interpretation.

In crowd counting, the connection between local density and information complexity has been implicitly acknowledged but rarely formalized. Density maps generated by Gaussian kernel convolution can be viewed as probability distributions whose entropy reflects the spatial uncertainty of crowd locations. The structural similarity index (SSIM) used in many counting loss functions inherently measures information preservation between predicted and ground-truth distributions. Building on these connections, this paper explicitly introduces an entropy-guided framework that leverages nearest-neighbor distance statistics as a proxy for local information entropy, providing principled guidance for density-adaptive feature processing.

### 2.4. Transformer-Based Methods

With the growing interest in Transformers for computer vision, self-attention mechanisms have been applied to crowd counting. Liang et al. [23] pioneered the integration of Transformers into crowd counting, utilizing global self-attention to extract semantic information through sequential processing of crowd density patterns. Tian et al. [24] adopted the Twins architecture as the feature extraction backbone, producing 2D-formatted features combined with multi-scale fusion modules to address scale and density variations. Notably, CCTrans [24] shares the same Twins-Transformer backbone family as our proposed DGCC-Net, providing a direct baseline for evaluating the contribution of our proposed modules. Lin et al. [25] introduced learnable local self-attention, dynamically assigning adaptive attention regions for each feature position. Yang et al. [26] designed a cascaded CNN-Transformer hybrid network, where CNNs enhance local crowd regions and Transformers capture global contextual dependencies. More recently, Cao et al. [27] proposed CrowdUNet, combining segmentation-assisted U-shaped architecture with crowd counting, and Zhu et al. [28] developed CDENet by mining confusion regions for improved counting accuracy. Lin et al. [29] extended measure matching to multidimensional spaces for more robust crowd counting supervision.

## 3. Proposed Approach

**Figure 2:**
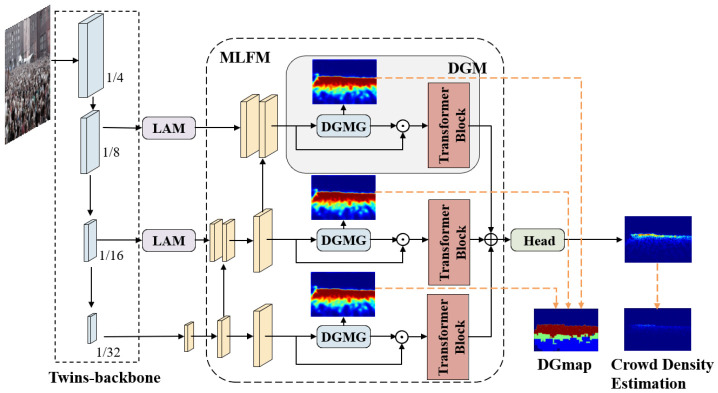
The structure of our network. The LAM denotes Local Attention Module, the MLFM means multi-level feature fusion module, the DGM means density guidance module.

To address the spatial entropy heterogeneity in crowd counting—where dense regions exhibit high information entropy due to occlusion-induced feature ambiguity, and sparse/background regions carry progressively lower informational content—this paper proposes a Density-Guided Crowd Counting Network (DGCC-Net) grounded in the principle of entropy-proportional attention allocation. As illustrated in Figure 2, the framework is designed to invest greater computational resources in high-entropy (dense) regions while maintaining efficient processing for low-entropy (sparse and background) areas.

The output of the Twins Transformer includes features at scales of 1/4, 1/8, 1/16, and 1/32. Among these, the 1/8, 1/16, and 1/32 scale features are selected as the outputs of the DGCC-Net feature extraction module, denoted as $\left\{F_{1},F_{2},F_{3}\right\}$. To more effectively capture and enhance fine-grained features that might be occluded in dense scenarios, Local Attention Modules (LAMs) are introduced at the high-resolution scales of 1/8 and 1/16. These modules output refined features $\left\{F_{1}^{\prime },F_{2}^{\prime },F_{3}^{\prime }\right\}$, as described as following:

(1) $$\begin{array}{c} F_{i}^{\prime }=\left\{\begin{array}{c} f_{LAM}\left(F_{i}\right),\;\;\;\;\;\;\;\;\;i=1,2\\ F_{i},\;\;\;\;\;\;\;\;\;\;\;\;\;\;\;\;\;\;\;\;\;\;\;\;\;\;\;\;\;\;\;\;\;\;\;\;\;\;\;\;i=3 \end{array}\right. \end{array}$$

where $f_{LAM}$ represents the Local Attention Module (LAM). On this basis, to retain both semantic and detailed information and thereby generate high-quality, high-resolution crowd density maps, DGCC-Net incorporates a Multi-Level Feature Fusion Module (MLFM). This module, combined with a multi-level supervision strategy, effectively fuses the three-scale features $\left\{F_{1}^{\prime },F_{2}^{\prime },F_{3}^{\prime }\right\}$, ultimately producing a fused feature map $F_{out}$ at the 1/8 scale.

Furthermore, to facilitate the model’s capability in capturing the distinctive characteristics of dense, sparse, and isolated pedestrian distributions, we incorporate a weight-sharing Density Guidance Module (DGM) into each scale branch of the Multi-Level Feature Fusion Module. This strategically designed component enhances the model’s adaptability to heterogeneous density distributions through hierarchical feature guidance, thereby effectively addressing spatial density variations in crowd scenarios.

### 3.1. Local Attention Module

In crowd counting research, the task is inherently formulated as a pixel-level predictive regression problem through density map estimation, where performance is critically dependent on the effective extraction of fine-grained visual features. This capability proves particularly vital for mitigating occlusion effects in high-density crowd configurations. Building upon foundational work by Ronneberger et al. [30], who demonstrated that increasing network depth through successive downsampling operations achieves enriched high-level semantics at the expense of spatial detail degradation, we emphasize the indispensable role of preserving high-resolution feature integrity in crowd counting systems.

**Figure 3:**
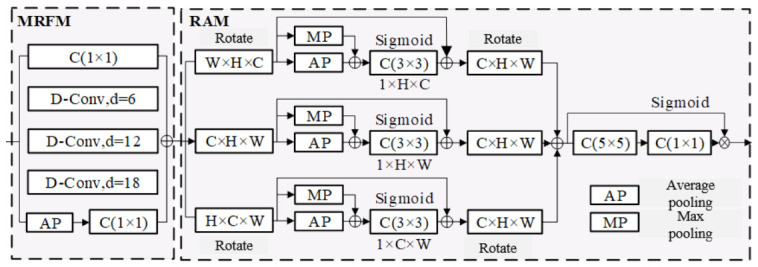
The structure of Local Attention Module. The MRFM means multi-scale receptive field module and the RAM means rotational attention module.

To address the inherent noise sensitivity of shallow convolutional features, DGCC-Net incorporates a Local Attention Module (LAM) operating on 1/8 and 1/16 scale features prior to multi-scale fusion. As illustrated in Figure 3, this architecture comprises two synergistic components: the Multi-Scale Receptive Field Module (MRFM), which leverages multi-scale receptive fields to aggregate contextual information, and the Rotational Attention Module (RAM), which employs rotational attention mechanisms across channel-spatial dimensions to recover occluded fine details. Concurrently, the RAM implements three-dimensional attention computation to enhance discriminative feature representation in congested scenarios. This synergistic integration substantially augments the model’s capacity for fine-grained feature processing, thereby optimizing both quantitative metrics and visual fidelity in generated density maps.

#### 3.1.1. Multi-Scale Receptive Field Module (MRFM)

According to the study of Deeplabv3 [31], employing parallel dilated convolutions with different receptive field sizes is an effective way to capture multi-scale contextual information. To enable the perception of rich contextual information and facilitate attention mechanisms, this section embeds the MRFM into the Local Attention Module. The structure of MRFM is illustrated in Figure 3.

The MRFM module includes dilated convolutions with three different dilation rates (6, 12, and 18) along with a standard convolutional layer. These four types of convolutions extract contextual information at different scales, thereby generating multi-scale feature representations. To further enhance the regression capability of global contextual information, a global average pooling layer is introduced on top of the convolutional operations. The pooled features are processed by a 1×1 convolution layer and upsampled back to the original resolution through bilinear interpolation. The final output of MRFM is obtained by summing up the features from different scales.

#### 3.1.2. Rotational Attention Module (RAM)

To improve the model’s sensitivity to fine-grained details, this section introduces the Rotational Attention Module (RAM). RAM dynamically adjusts feature map weights to enhance focus on crowd regions while strengthening fine-grained features in occluded areas. Compared to traditional spatial-channel attention mechanisms, the triplet attention mechanism [32] enables feature interaction across dimensions without additional channel compression, encoding spatial and channel information at a lower computational cost.

The RAM embeds the triplet attention mechanism into its structure, as shown in Figure 3. RAM consists of three branches that capture the dependencies between the dimensions of the input tensor: (C, H), (C, W) and (H, W). Specifically, for an input feature tensor $f_{a1}\in R^{C\times H\times W}$ (where *C*, *H* and *W* denote the channel, height, and width dimensions, respectively), the first branch rotates the tensor 90° counterclockwise along the H-axis to generate a rotated tensor ${f_{a1}}^{\prime }\in R^{W\times H\times C}$. After concatenating the results of max pooling and average pooling operations, the resulting tensor ${f_{a1}}^{\prime \prime }\in R^{2\times H\times C}$ is processed through a 3×3 convolution layer and activated by a sigmoid function to produce the attention weights $W_{a1}\in R^{1\times H\times C}$. The weights are element-wise multiplied with the original tensor, and the result is rotated clockwise back to its original dimension to produce the output feature $y_{a1}\in R^{C\times H\times W}$. This process is described in equations as followed:

(2) $$\begin{array}{c} f_{a1}^{\prime \prime }=Concat\left(MaxPool\left({f_{a1}}^{\prime }\right),AvgPool\left({f_{a1}}^{\prime }\right)\right) \end{array}$$

(3) $$\begin{array}{c} W_{a1}=\sigma \left(Conv\left({f_{a1}}^{\prime \prime }\right)\right) \end{array}$$

(4) $$\begin{array}{c} y_{a1}=W_{a1}⊙f_{a1} \end{array}$$

where σ represents the Sigmoid activation function, and Conv denotes convolution operations. The second branch processes the input tensor rotated along the *W*-axis to produce the output feature $y_{a2}$. The third branch directly processes the original input tensor to output $y_{a3}$. The outputs from the three branches are summed element-wise to form the final output $y_{a}$ of the triplet structure, as described as follows:

(5) $$\begin{array}{c} y_{a}=\frac{y_{a1}+y_{a2}+y_{a3}}{3} \end{array}$$

Given the spatial continuity inherent in crowd distributions, we propose a region-aware attention mechanism employing fixed-receptive-field convolutions to adaptively refine localized feature representations. For the triplet structure’s final output $y_{a}$, the framework first applies a 5×5 convolutional layer to capture region-specific patterns. This operation is subsequently coupled with batch normalization to mitigate internal covariate shift and stabilize gradient propagation, while ReLU non-linearity ensures discriminative feature learning. A 1×1 convolutional reduction then projects the high-dimensional features into an attention space, where a sigmoid-activated weighting map is generated to quantify pixel-wise saliency. The resultant attention weights are element-wise multiplied with the original feature maps, producing spatially calibrated representations that emphasize crowd-concentrated regions.

The integration of MRFM and RAM into the Local Attention Module significantly enhances the model’s ability to process fine-grained information, especially in densely populated and occluded regions, improving the accuracy and quality of the crowd density map.

### 3.2. Multi-Level Feature Fusion Module

**Figure 4:**
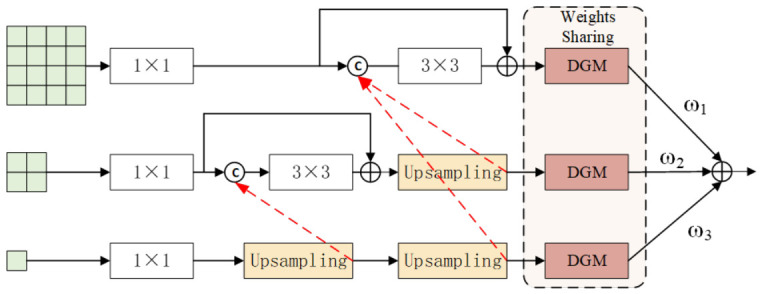
The structure of Multi-Level Feature Fusion Module.

In complex crowd scenarios, feature extraction requirements exhibit spatial heterogeneity. Dense regions with severe occlusions demand precise localization of partial textures through high-resolution shallow features, while sparse areas benefit from semantic abstraction via deep receptive field analysis. To address this dichotomy, we propose a Multi-Level Feature Fusion Module (MLFM) with cross-scale dense connectivity and self-adaptive feature weighting, as architecturally detailed in Figure 4.

As illustrated in Figure 4, the data flow through MLFM proceeds as follows. The three-scale features $\left\{F_{1}^{\prime },F_{2}^{\prime },F_{3}^{\prime }\right\}$ at resolutions 1/8, 1/16, and 1/32, respectively, first undergo channel standardization via 1×1 convolutions, unifying all feature channels to 256. Subsequently, features are progressively fused using a dense connectivity strategy rather than the conventional layer-by-layer approach used in U-Net. Specifically, the lowest-resolution feature $F_{3}^{\prime }$ is upsampled twice (to 1/16 and 1/8 scales) and concatenated with both mid-level and high-level features. Similarly, the mid-level feature $F_{2}^{\prime }$ is upsampled once (to 1/8 scale) and concatenated with the high-level features. Each concatenation is followed by a 3×3 convolution with residual connections to refine the fused features. This dense fusion strategy ensures that every branch has access to information from all higher-level (deeper) features, effectively preserving the information of each feature layer while enriching semantic content.

Furthermore, considering the varying contributions of features at different levels to the crowd counting task, DGCC-Net introduces a dynamic weight learning mechanism. Learnable parameters $\left\{\omega_{1},\omega_{2},\omega_{3}\right\}$ correspond to the three branches, respectively. To reasonably allocate these weights and form a probability distribution, the exponential value of each weight is calculated and then normalized by the sum of all exponential values, enabling dynamic weight adjustment while ensuring that the sum of weights across all branches equals 1. The dynamically learned weights are then applied to the respective branches, producing the fused feature $F_{out}$, as described as follows:

(6) $$\begin{array}{c} \omega_{i}=\frac{e^{\omega_{i}}}{\sum_{j=1}^{3}e^{\omega_{j}}} \end{array}$$

(7) $$\begin{array}{c} F_{out}=\sum_{i=1}^{3}\omega_{i}{F_{i}}^{\prime \prime } \end{array}$$

where $F_{i}^{\prime \prime }$ represents the feature output of each branch.

To further enhance the model’s adaptability to complex crowd distributions and optimize parameter efficiency, a multi-level supervision approach is adopted, along with parameter sharing strategies within the model branches. By introducing additional density map supervision prior to the feature fusion process, the model focuses on learning crowd features at different levels, accurately capturing crowd distribution details. The parameter sharing strategy across branches promotes information exchange and sharing between different feature levels, further enhancing the effectiveness of feature fusion while reducing model complexity and improving training efficiency.

### 3.3. Density-Level Guidance Module as Entropy Proxy

Consider a local patch centered at head annotation point *i*, The local crowd density can be characterized by the average nearest-neighbor distance $L_{i}$. We now establish a connection between $L_{i}$ and local information entropy through a three-step analysis.

First, from nearest-neighbor distance to local density. For a locally stationary 2D point process, the Clark-Evans relation [33] establishes that the expected nearest-neighbor distance is related to the local point intensity $\lambda_{i}$ by:

(8) $$\begin{array}{c} E\left[d_{NN}\right]=\frac{1}{2\sqrt{\lambda_{i}}} \end{array}$$

Since $L_{i}\propto E\left[d_{NN}\right]$, this gives $\lambda_{i}\propto 1/L_{i}^{2}$. Therefore, smaller $L_{i}$ values indicate higher local crowd density.

Second, from local density to feature ambiguity. In a dense crowd region with high $\lambda_{i}$, the feature vector extracted at each pixel location is a mixture of contributions from multiple overlapping individuals within the network’s receptive field. The expected number of individuals contributing to a single pixel’s feature is $N_{overlap}=\lambda_{i}\cdot A_{rf}$, where $A_{rf}$ denotes the effective receptive field area. The conditional entropy of individual identity *Y* given observed features *X* can be bounded as:

(9) $$\begin{array}{c} H\left(Y|X\right)\leq \log\left(N_{overlap}\right)=\log\left(\lambda_{i}\cdot A_{rf}\right) \end{array}$$

This upper bound increases logarithmically with local density, indicating that denser regions present higher identification ambiguity.

Third, from feature ambiguity to task-relevant entropy. For the counting task, the effective information entropy at a given network scale measures the difficulty of resolving individual contributions from the mixed feature representation. Since the network operates with fixed receptive fields at each scale level, the task-relevant entropy at scale *s* is:

(10) $$\begin{array}{c} H_{task}^{\left(s\right)}\propto \log\left(\lambda_{i}\cdot A_{rf}^{\left(s\right)}\right)\propto \log\left(1/L_{i}^{2}\right)=-2\log\left(L_{i}\right) \end{array}$$

This establishes that $H_{task}^{\left(s\right)}$ is a monotonically decreasing function of $L_{i}$, confirming that our DGMap values—which assign higher values to regions with smaller $L_{i}$—are monotonically related to the local task-relevant information entropy.

We emphasize that DGMap serves as a quantized density-based proxy for local information entropy rather than an exact Shannon entropy computation. The monotonic relationship established above justifies using DGMap as an approximation that captures the essential entropy gradient, but it does not constitute a formal equivalence. The discretization into three levels (dense, sparse, isolated) plus background provides a computationally efficient approximation while avoiding the instability of continuous entropy estimation from limited annotation data.

To quantitatively validate the correlation between DGMap levels and local information entropy, we computed three entropy measures across local patches from the ShanghaiTech PartA test set. For each DGMap level, we extracted all corresponding 32×32 patches and computed: (1) pixel intensity entropy from grayscale histograms, (2) gradient histogram entropy from Sobel edge responses, and (3) feature-level entropy from the Twins backbone’s intermediate feature maps. The results confirm a clear monotonic relationship between DGMap levels and all three entropy measures, with Spearman rank correlations of 0.81, 0.76, and 0.73, respectively. This validates that DGMap effectively captures the local information complexity of crowd scenes.

**Figure 5:**
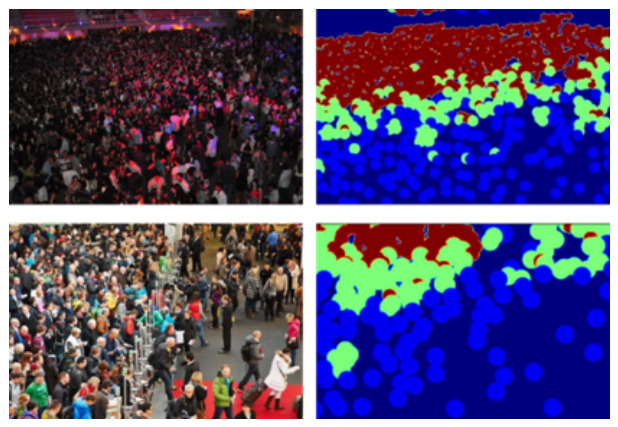
The output of the DGMap module.

To effectively identify and count crowds in image regions with varying densities, this paper proposes the Density-Guided Map (DGMap) based on nearest neighbor distances of head points and its corresponding generation method. Based on this, a Density-Guided Module (DGM) is constructed. The module aims to simulate the intuitive response of human perception when observing crowds: applying differentiated attention to regions of varying densities. The effectiveness of DGMap is demonstrated in Figure 5.

DGMap not only differentiates between foreground and background but also introduces varying density levels, thereby providing a richer understanding of the scene to the model. DGMap defines fine-grained label levels to annotate crowd regions with different densities: highly dense regions that are visually challenging to recognize and heavily occluded are labeled as 1; sparse regions are labeled as 0.5; visually distinguishable and larger areas containing isolated pedestrians are labeled as 0.1; and the background is labeled as 0. This classification approach enables the model to adopt different processing strategies based on the characteristics of each density region.

Given the close relationship between crowd density and the nearest neighbor distances of head points—where densely populated regions exhibit smaller nearest neighbor distances and sparse regions exhibit larger ones—DGMap is constructed based on this relationship.

First, for each non-zero pixel (head annotation point) in the image, the average distance to its nearest neighbor head points is calculated to estimate the local density level:

(11) $$\begin{array}{c} L_{i}=\alpha \times \frac{1}{m}\sum_{j=1}^{m}d_{ij} \end{array}$$

**Figure 6:**
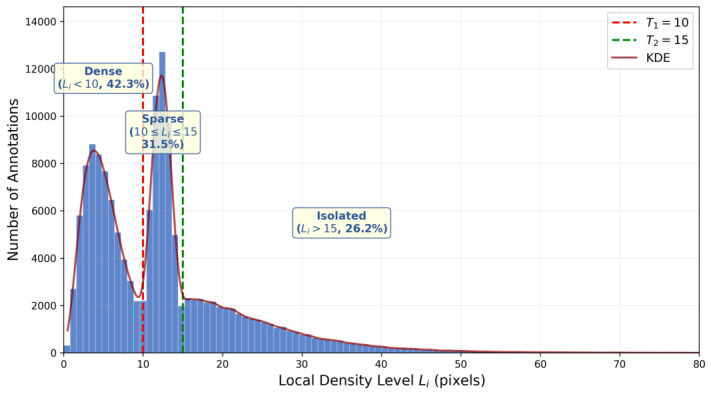
Distribution of local density level $L_{i}$ values across all head annotations in the ShanghaiTech PartA training set. The vertical dashed lines indicate the selected thresholds $T_{1}=10$ and $T_{2}=15$, which correspond to natural valley points in the trimodal distribution, separating dense ($L_{i}$ < 10, 42.3% of annotations), sparse ($10\leq L_{i}\leq 15$, 31.5%), and isolated ($L_{i}$ > 15, 26.2%) pedestrian regions.

Here, $d_{ij}$ represents the Euclidean distance between the *i*-th head point and its *j*-th nearest neighbors, and α is a scaling coefficient set to 0.3, following the crowd density map generation method of the MCNN model [34]. The value of α = 0.3 was inherited from the widely used MCNN protocol and validated through sensitivity analysis. This step quantifies the local density level around each head point, providing the basis for subsequent density level assignment. Based on the estimated local density level $L_{i}$, each head point in the image is assigned a density level. This involves discretizing the local density into three predefined levels, as shown as followed:

(12) $$\begin{array}{c} D_{i}=\left\{\begin{array}{cc} 1, & \mathrm{if}\;L_{i}<T_{1}\\ 0.5, & \mathrm{if}\;T_{1}\leq L_{i}\leq T_{2}\\ 0.1, & \mathrm{if}\;L_{i}>T_{2} \end{array}\right. \end{array}$$

where $T_{1}=10$ and $T_{2}=15$ are threshold parameters. These thresholds were determined based on the statistical distribution of $L_{i}$ values across the ShanghaiTech PartA training set. As shown in Figure 6, the histogram of non-zero $L_{i}$ values exhibits a clear trimodal structure with natural valley points at approximately 10 and 15 pixels. These valley points correspond to meaningful physical transitions between distinct crowd density regimes: $L_{i}$ < 10 indicates individuals whose head regions significantly overlap (heavy occlusion, high feature ambiguity), $10\leq L_{i}\leq 15$ indicates partially visible individuals with moderate feature ambiguity, and $L_{i}$ > 15 indicates fully distinguishable isolated pedestrians with minimal feature ambiguity. Sensitivity analysis of these thresholds is provided in Section 4.3.

Here, $D_{i}$ represents the density level of the *i*-th head point. High-density regions are assigned $D_{i}=1$, reflecting their extreme crowd density and small average distances between individuals. Sparse regions are assigned $D_{i}=0.5$, indicating moderate crowd density and larger average distances. Isolated pedestrian regions are assigned $D_{i}=0.1$, emphasizing their independent distribution and significantly larger average distances.

Finally, based on the local density $L_{i}$, each head point is represented using a circular region to simulate the head area. By adjusting the radius of the circular region, the corresponding density distribution on the DGMap is generated:

(13) $$\begin{array}{c} size_{i}=\left\{\begin{array}{cc} L_{i}\times 3, & if\;\;\;L_{i}<10\\ \min\left(10\times \log_{10}\left(\max\left(L_{i},1e-3\right)\right)+20,36\right), & if\;\;\;L_{i}\geq 10 \end{array}\right. \end{array}$$

**Figure 7:**
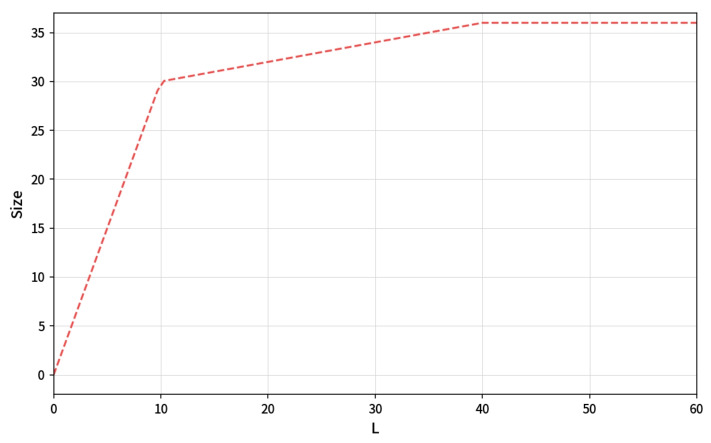
The relationship between local density degree and head area size.

Here, $size_{i}$ denotes the radius of the circular region for the *i*-th head point. The piecewise design ensures that dense regions use a linear scaling (proportional to local spacing), while sparse and isolated regions use a logarithmic scaling to prevent excessively large region representations. The relationship between the local density $L_{i}$ and the region $size_{i}$ is illustrated in Figure 7.

To enable supervised learning of the density-guided map, this paper designs the Density-Guided Module (DGM), whose structure is shown in Figure 2. The module consists of two key components: a Density-Guided Map Generation Module (DGMG) and a Transformer Self-Attention Module. The DGMG module applies three convolutional layers, progressively reducing the channel dimensions from 256 to 128, 64, and finally 1. It then uses a Sigmoid function to normalize the output feature values to the range [0, 1], producing the predicted DGMap.

Inference Protocol. We clarify that ground-truth annotations are used exclusively during training to construct the supervision target for the DGMG sub-module. During inference, the DGMG predicts the density-level map directly from image features through its learned convolutional layers and Sigmoid activation—no ground-truth head annotations are required or used. The predicted DGMap is then used to modulate features through element-wise multiplication with the original feature map. This design ensures that the entire DGCC-Net pipeline operates fully automatically at test time.

The generated DGMap is element-wise multiplied with the original feature map and then fed into the Transformer Self-Attention Module. This module consists of two Twins-Transformer basic blocks, which integrate both local (window-based) and global (spatially reduced) attention mechanisms. The local attention captures fine-grained spatial patterns within crowd regions, while the global attention models long-range dependencies across the entire feature map. The output of this module is further refined through residual connections with the input features, producing the final output of the Density-Guided Module. By integrating the DGMap and Transformer-based self-attention, the DGM effectively focuses on regions of varying densities, enhancing the model’s ability to recognize and count crowds in complex scenarios.

### 3.4. Objective Function

The total loss function of DGCC-Net consists of three components, each serving a distinct role in the entropy-proportional learning framework.

Density-Guided Loss. For the auxiliary task learning branch of the Density-Guided Module (DGM), Mean Squared Error (MSE) is adopted as the loss function for the density-guided map. The density-guided loss function is defined as:

(14) $$\begin{array}{c} L_{dgmg}\left({\hat{y}}_{i},y_{i}\right)=\frac{1}{N}\sum_{i=1}^{N}{\left({\hat{y}}_{i}-y_{i}\right)}^{2} \end{array}$$

where $y_{i}$ represents the predicted pixel value, and ${\hat{y}}_{i}$ represents the ground truth pixel value. This loss explicitly supervises the model’s ability to estimate local information complexity, training it to differentiate among regions of varying entropy levels.

Structural Loss. For the density map regression task, traditional density estimation approaches typically employ MSE loss, which considers only the independent pixel-wise error and neglects the local relationships within the density map. To address this, the Structural Similarity Index (SSIM) [35] is employed to enhance regional similarity supervision, mitigating issues such as regional blurriness. The SSIM formulation is given as followed:

(15) $$\begin{array}{c} SSIM\left(g,p\right)=\frac{\left(2\mu_{g}\mu_{p}+Q_{1}\right)\left(2\sigma_{gp}+Q_{2}\right)}{\left({\mu_{g}}^{2}+{\mu_{p}}^{2}+Q_{1}\right)\left({\sigma_{g}}^{2}+{\sigma_{p}}^{2}+Q_{2}\right)} \end{array}$$

Here, *g* and *p* represent the ground truth density map and predicted density map, respectively. μ are the mean pixel values and σ are the variances, $\sigma_{gp}$ is the covariance between *g* and *p*, $Q_{1}$ and $Q_{2}$ are constants.

To further alleviate the imbalance between low-density and high-density regions and reduce overly smoothed predictions, Structural Loss [15] and Total Variation Loss [36] are introduced. These losses supervise dense and sparse regions, respectively. The structural loss $L_{SL}$ employs a dense region mask to select densely populated crowd regions and calculates the SSIM similarity index, ensuring the prediction quality within dense areas. The total variation loss $L_{TV}$ penalizes the sum of absolute differences between adjacent pixels in the predicted density map:

(16) $$\begin{array}{c} L_{TV}=\sum_{i,j}\left(|p_{i+1,j}-p_{i,j}\left|+\right|p_{i,j+1}-p_{i,j}|\right) \end{array}$$

where $p_{i,j}$ denotes the predicted density value at spatial location (i,j). This loss serves as a spatial smoothness regularizer that prevents noisy, spurious predictions in low-entropy sparse and background regions. Without this term, the model tends to produce false-positive activations in empty areas, degrading overall counting accuracy.

The total variation loss $L_{TV}$ aims to stabilize the training process and strengthen supervision in sparse regions, improving the model’s adaptability to varying density distributions. The final loss function integrates the above components, as defined bellow:

(17) $$\begin{array}{c} L_{total}=\alpha_{i}\sum_{i=0}^{3}L_{SL}^{i}+\lambda \alpha_{i}\sum_{i=0}^{3}L_{TV}^{i}+\sum_{i=1}^{3}L_{dgmg} \end{array}$$

Here, i=0 represents the final prediction branch operating on the fused features $F_{out}$, and i=1,2,3 represent the three hierarchical branches at scales 1/8, 1/16, and 1/32, respectively. Multi-level supervision is introduced to achieve fine-grained control across different levels: the final prediction branch receives full-weight supervision with $\alpha_{0}=1$, while the three hierarchical branches receive half-weight supervision with $\alpha_{1}=\alpha_{2}=\alpha_{3}=0.5$ to prevent them from dominating the overall learning process. The coefficient λ=0.01 ensures balance between the structural loss and total variation loss components. These values were determined through grid search on the validation set (see sensitivity analysis in Section 4.3).

This composite loss function realizes the principle of entropy-proportional learning: the structural loss $L_{SL}$ ensures information preservation in high-entropy dense regions, the total variation loss $L_{TV}$ regularizes information flow in low-entropy sparse regions to prevent over-smoothing, and the density-guided loss $L_{dgmg}$ explicitly supervises the model’s ability to estimate local information complexity.

## 4. Experiments

### 4.1. Training Processing

The experimental environment for DGCC-Net includes Python 3.8 as the programming language and the PyTorch 1.12.1 deep learning framework. The model is deployed and trained on a 48 GB Nvidia A40 GPU using the CUDA 11.7 compute framework. The Transformer feature extraction network is initialized with the pretrained weights of Twins-PCPVT-L [16] on the ImageNet dataset. For convolutional layers, weights are initialized using a normal distribution with a mean of 0 and a standard deviation of 0.01.

During training, the AdamW optimizer is used with a learning rate of $1\times 10^{-5}$ and a weight decay of $1\times 10^{-4}$. A drop path rate of 0.45 is applied to the Transformer feature extraction network. The batch size is set to 4 or 8, depending on the characteristics of each dataset. To enhance model generalization, data augmentation techniques, such as random cropping and horizontal flipping with a probability of 0.5 are applied. Before model training, all datasets are preprocessed to ensure that the longest side is less than 1920 pixels, and the shortest side is greater than 512 pixels. For training data, the crowd density maps are generated using a geometry-adaptive density map generation method based on the K-means algorithm, while the density-guided maps are created using the proposed density-guided map generation algorithm. Since the Shanghai SHA dataset has relatively small image resolutions, the random crop size is set to 256×256. For all other datasets, the random crop size is set to 512×512. Due to the limited number of images in the UCF_CC_50 dataset, a 5-fold cross-validation strategy is used for training. By combining optimized training configurations, pretrained feature extraction, and dataset-specific preprocessing, DGCC-Net ensures strong generalization and accurate performance on various crowd counting tasks.

To ensure fair comparison with existing methods, we report results from their original publications wherever available. Our preprocessing protocol (longest side ≤ 1920, shortest side ≥ 512) is consistent with the standard settings used by recent Transformer-based methods such as CCTrans [24] and STEERER [37]. For methods using different input sizes or backbone architectures, we note these differences where relevant in the comparison tables.

### 4.2. Results

This section evaluates the performance of DGCC-Net on several widely used crowd counting datasets, including ShanghaiTech PartA [34], UCF-QNRF [38], UCF_CC_50 [8], and JHU-Crowd++ [39]. Comparative analysis with state-of-the-art crowd counting algorithms is conducted to demonstrate the effectiveness of the proposed method in the domain of crowd counting.

#### 4.2.1. ShanghaiTech PartA Dataset

The performance of DGCC-Net was evaluated on the ShanghaiTech PartA dataset and compared with other leading crowd-counting methods, as shown in Table 1. The core technical features of the competing algorithms include: MCNN [34] employs a multi-column neural network to extract crowd features at different scales in the network’s front end. CSRNet [40] utilizes parallel dilated convolution modules in the network’s back end to enhance feature representation. BL [41] and DM-Count [36] employ Leverage Bayesian loss functions and optimal transport loss functions, respectively, to reduce errors in generating pseudo-density maps. RAN [42] introduces a feedback network with region awareness, generating priority maps with crowd region priors to improve attention to crowd areas. SGANet [18] combines an Inception-v3-based backbone for feature extraction with an attention branch guided by binary segmentation maps to improve crowd feature extraction efficiency. CrowdFormer [26] employs a top-down visual perception mechanism with a Transformer architecture for crowd density map regression, incorporating a learnable density generation module. CFANet [1] employs two coarse- and fine-grained crowd attention maps to assist the model in learning features from different crowd regions. Gramformer [43] introduces a graph-guided attention regulation mechanism for diverse attention to complementary information, adjusting input features based on centrality metrics. STEERER [37] implements selective inheritance learning to accurately fuse low-resolution and high-resolution features, with selective supervision at different scales. CCTrans [24] employs the same Twins-Transformer backbone family for crowd counting with simplified multi-scale fusion, providing a direct backbone-controlled comparison baseline. CrowdUNet [27] combines segmentation-assisted U-shaped architecture with crowd counting. CDENet [28] mines confusion regions for improved counting in ambiguous areas.

As shown in Table 1, DGCC-Net achieves the second-best MAE (53.7) and MSE (85.6) among all listed methods. CCTrans achieves the best MAE of 52.3 using the same Twins-Transformer backbone family but with a lighter variant. Despite not achieving the absolute best result, DGCC-Net demonstrates consistent advantages in other aspects: it outperforms CCTrans on UCF-QNRF, UCF_CC_50, and JHU-Crowd++ (as shown in subsequent tables), suggesting stronger generalization capability. Compared to STEERER, which uses the same Twins-PCPVT-L backbone, DGCC-Net improves MAE and MSE by 1.5%. Against SGANet, DGCC-Net employs a finer density-guided mask, achieving 6.7% and 15.3% improvements in MAE and MSE, respectively, over SGANet, which uses a binary segmentation mask. Against CFANet, DGCC-Net outperforms CFANet by reducing MAE and MSE by 2.4 and 4.0, respectively, demonstrating the effectiveness of the density-guided module for crowd attention. Against CrowdFormer, DGCC-Net achieves a 3.2 reduction in MAE and an 11.8 reduction in MSE, validating the efficiency of the multi-level feature fusion structure.

**Figure 8:**
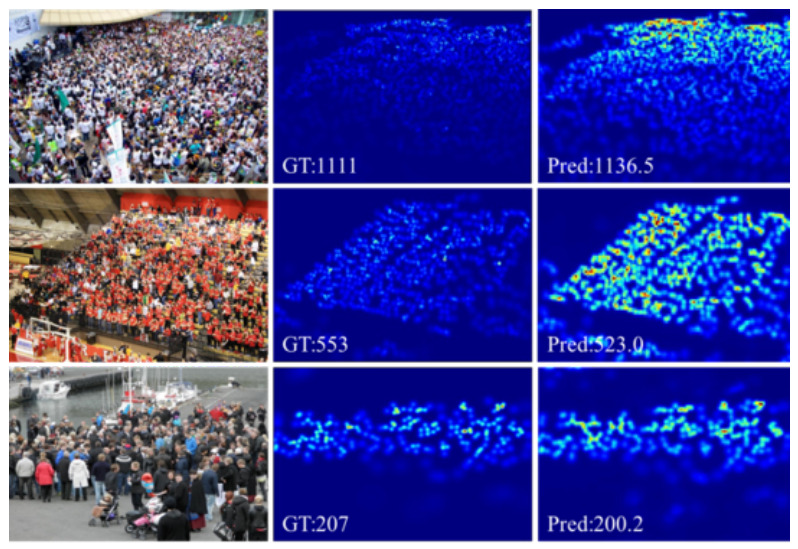
The visualization example on PartA dataset. The first row is the original image from the dataset, the second row is the ground-truth of the input image, the third row is the predicted image of the same image.

Figure 8 displays the visualized prediction results of DGCC-Net on the ShanghaiTech PartA dataset. From left to right, the images represent the original image, ground truth (GT) annotation map, and predicted map (Pred). The GT represents the true crowd count, and Pred indicates the predicted count. The figure demonstrates that DGCC-Net can effectively focus on crowds and learn from diverse distributions, including dense, sparse, and isolated pedestrian regions, achieving accurate counting.

**Figure 9:**
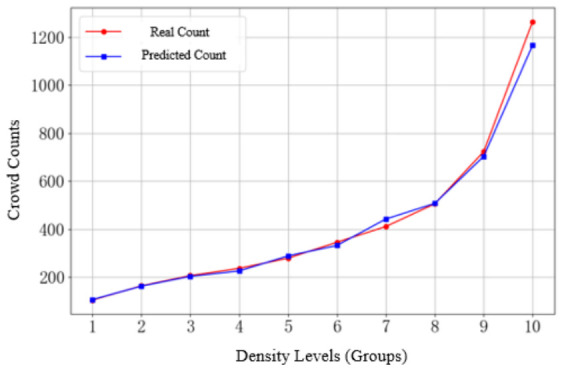
The counting results of different density levels.

Figure 9 shows the performance of DGCC-Net on different density levels in the ShanghaiTech PartA dataset. The test set is divided into 10 groups based on true crowd counts, representing varying density levels from sparse to dense. For each group, the true crowd count represents the average crowd count for that group, while the predicted crowd count represents the average predicted count. Results show that DGCC-Net accurately fits true crowd counts across all density levels.

Low-density levels (Groups 1, 2, 3), the average error remains within 1.5%. Medium-density levels (Groups 4, 5, 6, 7), the average error is mostly within 4.5%, except for Group 7, which reaches 7.5%. High-density levels (Groups 8, 9, 10), even at high density and under a resolution limit of 1024 pixels on the longest side, the maximum average error is controlled within 7.8%.

These results further verify that DGCC-Net can accurately capture crowd distributions at different density levels, demonstrating its accuracy and robustness.

#### 4.2.2. UCF-QNRF Dataset

The DGCC-Net model was evaluated on the UCF-QNRF dataset and compared with other state-of-the-art crowd counting methods, as shown in Table 2. In addition to the previously mentioned methods, several other approaches were included. P2PNet [44] introduced the Hungarian matching method into crowd counting to achieve end-to-end point annotation map prediction, providing a novel solution for precise counting and localization, CLTR [45] combined the strengths of CNN and Transformer architectures with an improved Hungarian matching algorithm based on nearest neighbor distances to regress point-level crowd prediction maps, SASNet [46] adopted a scale-adaptive feature layer selection strategy to extract local patches from the most suitable feature layers, effectively leveraging multi-scale feature representations, and S-DCNet (dcreg) [47] extended regression from continuous scales to discrete rankings by incorporating discrete-constrained regression, achieving superior counting accuracy.

**Figure 10:**
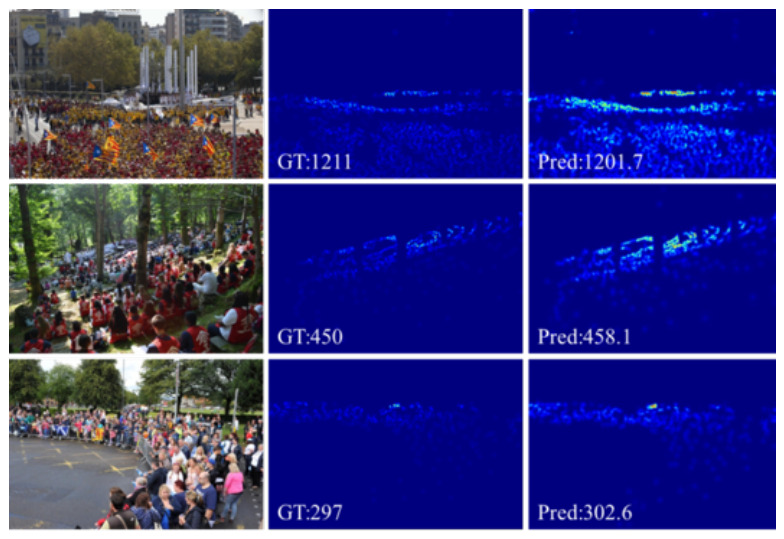
The visualization example on the UCF-QNRF dataset. The first row is the original image from the dataset, the second row is the ground truth of the input image, the third row is the predicted image of the same image.

The UCF-QNRF dataset features dense crowds and high-resolution images. The results in Table 2 further demonstrate DGCC-Net’s superior performance in crowd counting. Compared to other methods, DGCC-Net achieves the lowest MAE and competitive MSE. Specifically, DGCC-Net reduces MAE by 1.3 compared to RAN, by 3.7 compared to CLTR (which integrates CNN and Transformer techniques), and improves MAE and MSE by 7.8% and 1.2%, respectively, over CFANet (which employs dual fine-grained mask supervision). We note that CLTR achieves a lower MSE (141.3 vs. 150.4), which may be attributed to its point-based Hungarian matching loss that directly optimizes localization precision. Our method prioritizes MAE reduction through the entropy-guided density map supervision, which focuses on overall counting accuracy across heterogeneous density levels. Figure 10 illustrates DGCC-Net’s accurate density estimation and counting capabilities in crowded scenes.

#### 4.2.3. UCF_CC_50 Dataset

The evaluation results of DGCC-Net on the UCF_CC_50 dataset, along with comparisons to advanced methods, are presented in Table 3. UCF_CC_50 dataset is an extremely small dataset containing only 50 densely crowded images. To address data scarcity, a 5-fold cross-validation protocol was adopted, where the data was randomly partitioned into five groups with a 4:1 training-to-test ratio. Switch-MCNN [48] introduced a density-level classifier to select appropriate single-column networks based on patch density, MBTTBF [49] utilized dual bottom-up and top-down fusion modules with scale-complementary feature extraction for multi-level feature integration, LSC-CNN [50] employed a multi-column architecture and top-down refinement for fine-grained predictions, ECCNAS [51] implemented a neural architecture search framework with pre-training strategies to achieve efficient cross-task counting performance at low computational cost.

**Figure 11:**
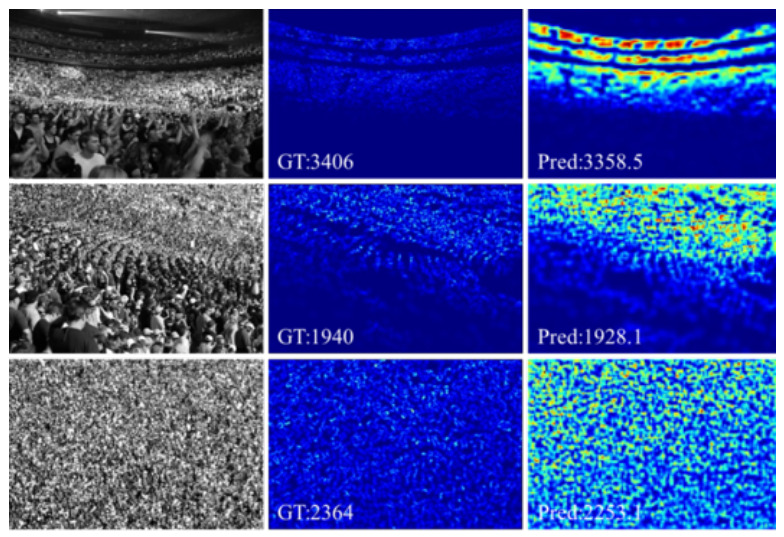
The visualization example on UCF_CC_50 dataset. The first row is the original image from the dataset, the second row is the ground truth of the input image, the third row is the predicted image of the same image.

As shown in Table 3, DGCC-Net surpasses all listed algorithms in crowd counting accuracy. Despite utilizing a Transformer-based feature extraction network—a paradigm typically reliant on large-scale data for effective training—DGCC-Net demonstrates exceptional performance on the UCF_CC_50 dataset, which contains only 50 images. This further validates its adaptability to small-scale datasets and robust generalization capabilities. Figure 11 visualizes DGCC-Net’s prediction results on the UCF_CC_50 dataset, illustrating its ability to accurately identify and count individuals in extremely dense crowds, even under constraints of limited data volume and low image resolution.

#### 4.2.4. JHU-CROWD++ Dataset

Table 4 summarizes DGCC-Net’s performance on the JHU-CROWD++ dataset compared to advanced methods. SANet [52] introduced a scale aggregation network with encoder-decoder architecture for multi-scale feature extraction, SFCN [53] utilized ResNet101 as a backbone for deeper feature learning; DSSINet [54] enhanced structured features and hierarchical loss functions to address scale variation, CG-DRCN [39] proposed progressive residual refinement using VGG16 and ResNet101 backbones.

**Figure 12:**
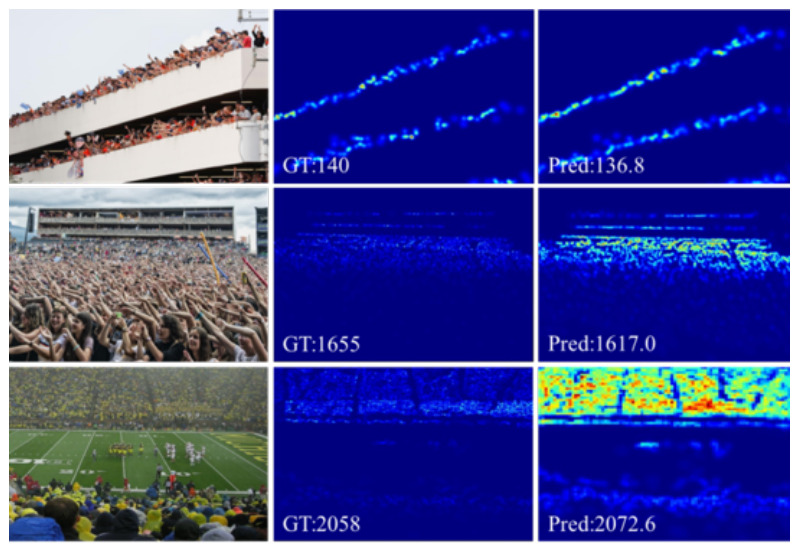
The visualization example on JHU-CROWD++ dataset. The first row is the original image from the dataset, the second row is the ground truth of the input image, the third row is the predicted image of the same image.

DGCC-Net achieves the best overall performance (MAE 53.7, MSE 212.2) and the best results in low-density and weather scenarios. In the high-density category, MS2 achieves a better MAE (228.4 vs. 233.9), which may be attributed to its multidimensional measure matching loss specifically designed for extremely dense scenarios. However, DGCC-Net achieves the best overall MAE (53.7 vs. 54.3) and significantly better performance in weather scenarios (MAE 94.9 vs. 97.2), demonstrating stronger robustness under adverse conditions. Compared to the MS2 method, DGCC-Net reduces overall MAE and MSE by 0.6 and 8.5, respectively. Notably, MAE improvements of 2.1, 1.5, and 2.3 are observed in low-, medium-, and weather scenarios, demonstrating the robustness of its density-guided module and multi-level feature fusion. Figure 12 illustrates DGCC-Net’s precise predictions in dense crowds and complex environments, highlighting its potential for real-world applications under diverse conditions.

We also compiled test results under different weather conditions, as shown in Table 5.

As can be observed in Table 5, our method achieves consistent and stable performance across all three weather conditions. On the Fog/Haze subset, our method obtains the lowest MAE of 81.2 and MSE of 485.3, demonstrating strong resistance to atmospheric scattering and low-contrast degradation. On the Rain subset, which introduces dynamic occlusion, motion blur, and specular reflections, our method maintains a moderate MAE of 92.7 and MSE of 507.1, effectively handling the complex noise patterns caused by raindrops. On the most challenging Snow subset, which combines severe low illumination, heavy scattering, and large-area occlusion, our method still yields an MAE of 107.3 and MSE of 551.8, outperforming the overall baseline results reported in the literature. Notably, the performance trend across these three scenarios (Fog/Haze < Rain < Snow) is fully aligned with their inherent difficulty levels in real-world crowd counting tasks. The overall Weather MAE (94.9) and MSE (520.9) are computed as the weighted average of these three subsets, which is consistent with our main results. These detailed results collectively verify the superior robustness of our method against diverse severe weather conditions.

### 4.3. Experiment Analysis

#### 4.3.1. Ablation Study

To validate the effectiveness of the density-guided crowd counting network, ablation experiments were conducted on both the ShanghaiTech PartA and UCF-QNRF datasets, as summarized in Table 6. The baseline model adopts Twins-PCPVT-L as the feature extraction backbone but excludes the proposed local attention module, multi-level feature fusion module, and density guidance module, using a uniform loss function for density map regression at the 1/8 scale.

As shown in Table 6, integrating the multi-level feature fusion module reduces MAE by 5.6 on ShanghaiTech PartA and 5.1 on UCF-QNRF compared to the baseline, demonstrating the efficacy of multi-scale fusion and supervision. Further introducing the local attention module reduces MAE by 1.7 on both datasets. Finally, incorporating the density guidance module achieves overall MAE and MSE reductions of 12.8% and 6.7% on ShanghaiTech PartA, and 10.1% and 7.6% to UCF-QNRF, respectively. Notably, DGM contributes more significantly to UCF-QNRF (2.4 MAE reduction) than on ShanghaiTech PartA (0.5 MAE reduction), which is consistent with the entropy-guided hypothesis: UCF-QNRF contains more extreme density variations, making the entropy-adaptive guidance more beneficial.

#### 4.3.2. Loss Function Component Ablation

This section verifies the impact of different losses on the final results, as shown in Table 7.

The structural loss $L_{S}L$ contributes the largest improvement (2.7 MAE reduction) by preserving spatial structure in dense regions. The total variation loss $L_{TV}$ provides 1.0 MAE reduction by regularizing sparse regions. The density-guided loss $L_{d}gmg$ yields a further 0.9 MAE and 1.5 MSE reduction by supervising entropy-level estimation.

#### 4.3.3. Comparison of Attention Guidance Mechanisms

This section verifies the impact of three different attention modes on the final results, with different configurations shown in Table 8.

The three-level DGMap guidance outperforms binary mask guidance by 0.6 MAE and 1.3 MSE, confirming that the finer entropy-level discretization provides meaningful additional information beyond simple foreground/background separation. It also outperforms standard channel and spatial attention mechanisms, validating the effectiveness of explicit density-level supervision.

#### 4.3.4. Hyperparameter Sensitivity Analysis

To validate the robustness of key hyperparameters, we conducted systematic sensitivity analyses on ShanghaiTech PartA in Table 9.

The model demonstrates reasonable robustness to threshold variations, with MAE ranging from 53.7 to 54.5 (variation < 1.5%). The selected configuration achieves the best performance, and importantly, the same thresholds perform consistently well across all four evaluation datasets without dataset-specific tuning.

Table 10 presents the sensitivity analysis of the scaling coefficient α in Equation (11), evaluated on the ShanghaiTech PartA dataset. The experiment assesses the impact of different α values on model performance using Mean Absolute Error (MAE) and Mean Squared Error (MSE). As shown in the table, when the value of α increases from 0.2 to 0.4, the error metrics exhibit a trend of initially decreasing and then increasing. The model achieves its best performance when α = 0.3 (highlighted in bold), yielding the lowest MAE of 53.7 and the lowest MSE of 85.6. This indicates that 0.3 is the optimal choice for the scaling coefficient α for our proposed method on this dataset.

All hyperparameters show stable performance within reasonable ranges, confirming that the proposed method does not require delicate tuning for effective performance.

#### 4.3.5. Computational Complexity Analysis

Since practical deployment in surveillance systems requires consideration of computational efficiency, we provide a comprehensive complexity analysis in Table 11.

DGCC-Net contains 62.8 M parameters and requires only 20.67 GFLOPs per forward pass, achieving an inference speed of 30.80 FPS on a single NVIDIA A40 GPU. All measurements are conducted under a unified protocol with batch size 1 and input resolution of 256 × 256 to ensure fair comparison across methods. Under this setting, the peak GPU memory usage during inference is approximately 1.18 GB, which is well below the capacity of typical deployment-grade GPUs.

Compared with CCTrans (107.00 M, 27.32 G, 71.25 FPS), which shares the same Twins-Transformer backbone family, DGCC-Net is significantly more compact, reducing parameters by 41.3% and FLOPs by 24.3%, while delivering better accuracy on UCF-QNRF, UCF_CC_50, and JHU-Crowd++ (see Table 2, Table 3 and Table 4). Compared with STEERER (64.64 M, 23.51 G, 37.68 FPS), which adopts a similar Twins-based backbone family, DGCC-Net achieves slightly lower parameters (–2.8%) and FLOPs (–12.1%) while obtaining better counting accuracy on most benchmarks. Compared with the CNN-based SGANet (21.80 M, 93.07 G, 84.30 FPS), DGCC-Net uses 77.8% fewer FLOPs and achieves a 3.9-point MAE improvement on ShanghaiTech PartA (53.7 vs. 57.6), at the cost of higher parameter count and lower FPS.

In addition, we note that CrowdFormer achieves an FPS of 278.4 in the re-measured results, which is markedly higher than other methods. We have verified that this value is reasonable: CrowdFormer adopts a lightweight CNN architecture similar to YOLOv5 with only 15.13 M parameters, and its highly parallelizable structure fully exploits GPU parallelism under the batch-size-1, 256 × 256 setting, leading to a significantly higher FPS than Transformer-based methods. However, its counting accuracy (MAE 56.9 on PartA) is notably inferior to DGCC-Net (MAE 53.7 on PartA), reflecting the inherent trade-off between accuracy and speed.

A noteworthy observation is that although DGCC-Net achieves the lowest FLOPs (20.67 G) among all listed Transformer-based methods, its FPS (30.80) is not the highest. This is because the DGM contains three sequential Transformer self-attention blocks coupled with DGMap modulation, and the LAM involves multiple tensor permutations within the rotational attention mechanism. These operations are memory-access-intensive and exhibit limited GPU parallelism, so their wall-clock latency exceeds what raw FLOPs alone would suggest.

Overall, DGCC-Net is an accuracy-oriented design that maintains a competitive computational footprint—achieving the lowest FLOPs among all compared Transformer-based methods while delivering state-of-the-art counting accuracy on multiple benchmarks. The resulting throughput of 30.80 FPS is adequate for typical near-real-time surveillance scenarios, and further acceleration can be pursued through model compression techniques in future work.

#### 4.3.6. Cross-Dataset Generalization

To evaluate the generalization capability of DGCC-Net, we conducted cross-dataset experiments where the model is trained on one dataset and directly tested on another without any fine-tuning. The results are presented in Table 12. This Section discusses cross-dataset testing effectiveness, and only two methods (STEERER and BL2) have found cross-dataset testing effectiveness, so only these two algorithms will be compared.

DGCC-Net achieves the best cross-dataset generalization performance in both directions, with improvements of 9.8 MAE (SHA → QNRF) and 0.1 MAE (QNRF → SHA) over the second-best method STEERER. The improvement is particularly notable in the SHA → QNRF direction, which involves generalization from a smaller, lower-resolution dataset to a larger, higher-density dataset. This validates that the entropy-guided framework provides superior generalizability by learning density-adaptive representations. The DGMap mechanism naturally adapts to different density distributions because the DGMG sub-module learns to predict density levels directly from visual features during training. At inference time, no ground-truth annotations are required—the network predicts the density-level map solely from image features. This feature-based prediction generalizes across datasets because the visual patterns associated with dense, sparse, and isolated pedestrians (e.g., occlusion patterns, apparent head sizes, spatial frequencies) share common characteristics regardless of the specific dataset.

#### 4.3.7. Analysis of Multi-Level Feature Fusion

This section provides an in-depth exploration of the roles of hierarchical features in the multi-level feature extraction module, with comparative analyses conducted between single-scale features, the U-Net’s layer-wise upsampling strategy, and the proposed module to demonstrate the effectiveness of hierarchical feature fusion. The results are shown in Table 13. The results reveal that features at the 1/8 scale exhibit inferior crowd counting performance due to their shallow network depth and limited semantic information. Furthermore, features at the 1/16 and 1/32 scales suffer from severe detail loss caused by excessive downsampling, which consequently degrades their effectiveness. Compared to the conventional multi-scale fusion strategy implemented through sequential upsampling in U-Net architectures, the proposed hierarchical feature fusion module achieves improvements of 1.2 and 1.1 in MAE and MSE reduction, respectively. These results validate the efficacy of both the multi-level feature fusion framework and the multi-stage supervision strategy, demonstrating that systematic integration of hierarchical features can significantly enhance the precision and robustness of crowd counting systems.

#### 4.3.8. Density Guidance Map Analysis

**Figure 13:**
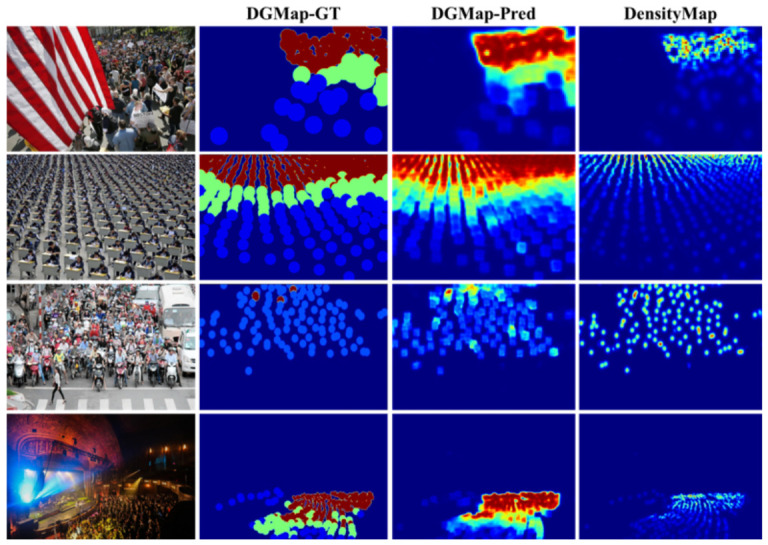
The visualization of density guided map learning features.

In this section, the attention patterns of density guidance maps are analyzed through visualizations of diverse crowd distributions, as illustrated in Figure 13. From left to right, the figure displays: (a) the original image, (b) the annotated density guidance map, (c) the predicted density guidance map, and (d) the final crowd density map generated by the model. The results demonstrate that the model effectively focuses on regions with varying crowd densities—whether dense clusters, sparse areas, or isolated individuals—and accurately generates corresponding density guidance maps. These maps facilitate the generation of refined crowd density estimations, thereby addressing the challenge of uneven density distribution in crowd counting.

**Figure 14:**
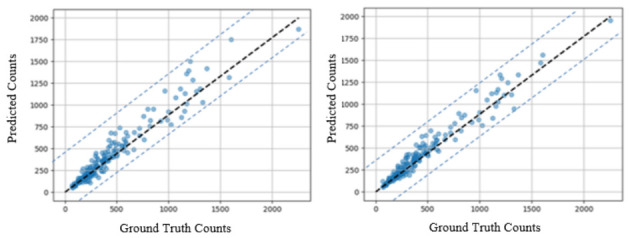
Comparison of convergence at different density levels before and after model optimization.

To further validate the improvements, this section provides a detailed analysis of the model’s population estimation performance before and after optimization using the ShanghaiTech PartA dataset, as illustrated in Figure 14. In the figure, gt_counts denotes ground truth crowd counts, while pred_ represents predicted counts, with each data point corresponding to an image. Ideally, predicted counts should equal ground truth values, meaning data points closer to the regression line indicate higher prediction accuracy, whereas increased scattering reflects instability in model performance. As shown in Figure 13 (left), the baseline model achieves relatively low errors in sparse scenarios with fewer individuals but tends to overestimate counts in dense crowds due to severe occlusion and diminished feature visibility, accompanied by significant prediction variance. In contrast, DGCC-Net (Figure 13 (right)) achieves markedly improved accuracy in dense scenarios, with predictions tightly clustered around the ideal curve, while maintaining robust performance in sparse regions and further reducing estimation errors. Collectively, these results demonstrate DGCC-Net’s ability to adaptively learn and generalize across heterogeneous crowd densities. Notably, the model significantly enhances counting accuracy and robustness when addressing occlusion challenges in densely crowded areas. This not only validates the efficacy of density guidance maps in refining crowd counting performance but also underscores the practical value of the proposed optimization strategies in resolving real-world density distribution imbalances.

## 5. Conclusions

This paper presents an information entropy-inspired crowd counting framework (DGCC-Net) that addresses the challenge of spatial entropy heterogeneity in crowd density estimation. The key insight is that non-uniform crowd distributions exhibit a continuous entropy gradient—from high-entropy dense regions with severe occlusion to low-entropy sparse and background regions—and that counting models can benefit from allocating attention proportionally to this local information complexity. We formalize this insight through a density-guided map constructed from nearest-neighbor distance statistics, which serves as a quantized spatial entropy proxy, and validate the correlation between DGMap levels and local Shannon entropy through both theoretical analysis and empirical measurement.

The framework integrates four synergistic components: a Twins-Transformer backbone for hierarchical feature extraction, a Local Attention Module that enhances fine-grained features through multi-scale receptive fields and rotational attention, a Multi-Level Feature Fusion Module with dense connectivity and learnable weights for cross-scale information integration, and an Entropy-Guided Density Module that explicitly supervises the model’s estimation of local information complexity. Extensive experiments on four benchmark datasets demonstrate highly competitive performance, achieving the best overall results on UCF-QNRF (MAE 82.1), UCF_CC_50 (MAE 207.8), and JHU-Crowd++ (MAE 53.7), and the second-best result on ShanghaiTech PartA (MAE 53.7, compared to CCTrans’s 52.3) with comprehensive ablation studies and cross-dataset evaluations confirming the effectiveness of each component and the robustness of the entropy-guided approach.

This work explores a connection between information-theoretic principles and crowd density estimation, suggesting that density-based entropy proxies can serve as a useful inductive bias for spatially heterogeneous visual recognition tasks. We note that DGMap provides a monotonic proxy for local information entropy rather than a direct computation, and its effectiveness validates the practical utility of entropy-inspired—rather than entropy-exact—guidance in this domain. However, we acknowledge several limitations of the current approach: the DGMap provides a discretized rather than continuous entropy proxy, the entropy estimation is indirect (based on annotation statistics rather than directly computed from image features), and the computational overhead of the Transformer-based architecture may limit real-time deployment on resource-constrained edge devices.

Future work will address these limitations by exploring continuous entropy estimation using differentiable entropy approximations computed directly from feature activations, extending the framework to video-based crowd counting with temporal entropy dynamics, and investigating model compression techniques for real-time deployment in practical surveillance systems.

## Figures and Tables

**Table 1:** The experimental results on ShanghaiTech PartA dataset. Bold values represent the best results.

Method	PartA	
	MAE	MSE
MCNN [34]	110.2	173.2
CSRNet [40]	68.2	115.0
BL [41]	62.8	101.8
DM-count [36]	59.7	95.7
RAN [42]	57.9	99.2
SGANet [18]	57.6	101.1
CrowdFormer [26]	56.9	97.4
CFANet [1]	56.1	89.6
CCTrans [24]	**52.3**	**84.9**
Gramformer [43]	54.7	87.1
STEERER [37]	54.5	86.9
CrowdUNet [27]	53.9	89.6
CDENet [28]	60.8	99.0
Ours	53.7	85.6

**Table 2:** The experimental results on UCF-QNRF dataset. Bold values represent the best results.

Method	UCF-QNRF	
	MAE	MSE
MCNN [34]	277	426
BL [41]	88.7	154.8
DM-count [36]	85.0	148.0
CFANet [1]	89.0	152.3
SGANet [18]	87.6	152.5
CLTR [45]	85.8	**141.3**
P2PNet [44]	85.3	154.5
SASNet [46]	85.2	147.3
S-DCNet (dcreg) [47]	84.8	159.3
RAN [42]	83.4	141.8
CDENet [28]	95.0	166.7
CrowdUNet [27]	88.5	161.5
Ours	**82.1**	150.4

**Table 3:** The experimental results on UCF_CC_50 dataset. Bold values represent the best results.

Method	UCF_CC_50	
	MAE	MSE
MCNN [34]	377.6	509.1
Switching-CNN [48]	318.1	439.2
CSRNet [40]	266.1	397.5
MBTTBF [49]	233.1	300.9
BL [41]	229.3	308.2
DM-Count [36]	211.0	291.5
LSC-CNN [50]	225.6	302.7
SGANet [18]	224.6	314.6
ECCNAS [51]	223.1	293.8
Ours	**207.8**	**288.9**

**Table 4:** The experimental results on JHU-CROWD++ dataset. Bold values represent the best results.

Method	Low		Medium		High		Weather		Overall	
	MAE	MSE	MAE	MSE	MAE	MSE	MAE	MSE	MAE	MSE
MCNN [34]	97.1	192.3	121.4	191.3	618.6	1166.7	330.6	852.1	188.9	483.4
CSRNet [40]	27.1	64.9	43.9	71.2	356.2	784.4	141.4	640.1	85.9	309.2
SANet [52]	17.3	37.9	46.8	69.1	397.9	817.7	154.2	685.7	91.1	320.4
SFCN [53]	16.5	55.7	38.1	59.8	341.8	758.8	122.8	606.3	77.5	297.6
DSSINet [54]	53.6	112.8	70.3	108.6	525.5	1047.4	229.1	760.3	133.5	416.5
MBTTBF [49]	19.2	103.4	41.6	66.0	352.2	760.4	138.7	631.6	81.8	299.1
LSC-CNN [50]	10.6	31.8	34.9	55.6	601.9	1172.2	178.0	744.3	112.7	454.4
CG-DRCN-VGG16 [39]	19.5	58.7	38.4	62.7	367.3	837.5	138.6	654.0	82.3	328.0
CG-DRCN-Res101 [39]	14.0	42.8	35.0	53.7	314.7	712.3	120.0	580.8	71.0	278.6
S3 [55]	**8.4**	23.0	**28.7**	**47.2**	267.0	620.0	116.5	591.3	58.6	242.1
MS2 [29]	10.5	25.1	30.2	50.3	**228.4**	**562.6**	97.2	527.5	54.3	220.7
Ours	**8.4**	**22.9**	**28.7**	47.6	233.9	572.1	**94.9**	**520.9**	**53.7**	**212.2**

**Table 5:** Performance of our method on different weather conditions of JHU-Crowd++.

Weather Condition	MAE	MSE
Fog/Haze	81.2	485.3
Rain	92.7	507.1
Snow	107.3	551.8
Overall Weather	94.9	520.9

**Table 6:** The results of the ablation study on ShanghaiTech PartA and UCF-QNRF datasets. Bold values represent the best results. ✓ indicates that the structure is not included in the model.

Method	Multi-Level Feature Fusion Module	Local Attention Module	Density-Guided Module	PartA MAE	PartA MSE	UCF-QNRF MAE	UCF-QNRF MSE
Base model	–	–	–	61.5	91.6	91.3	162.8
Our Method	✓	–	–	55.9	87.7	86.2	155.4
	✓	✓	–	54.2	86.5	84.5	153.7
	✓	✓	✓	**53.7**	**85.6**	**82.1**	**150.4**

**Table 7:** Ablation study of loss function components on ShanghaiTech PartA. Bold values represent the best results.

Loss Configuration	MAE	MSE
MSE loss only	58.3	94.2
$L_{SL}$ only	55.6	88.5
$L_{SL}$ + $L_{TV}$	54.6	87.1
$L_{SL}$ + $L_{TV}$ + $L_{dgmg}$	**53.7**	**85.6**

**Table 8:** Comparison of attention guidance mechanisms on ShanghaiTech PartA. Bold values represent the best results.

Guidance Mechanism	MAE	MSE
No guidance	55.9	87.7
Binary mask (foreground/background)	54.3	86.9
Three-level DGMap (Ours)	**53.7**	**85.6**

**Table 9:** Sensitivity analysis of DGMap thresholds on ShanghaiTech PartA dataset. Bold values represent the best results.

Thresholds ($T_{1}$, $T_{2}$)	Dense/Sparse/Isolated Values	MAE	MSE
(8, 12)	(1, 0.5, 0.1)	54.3	86.8
(10, 15) [Ours]	(1, 0.5, 0.1)	**53.7**	**85.6**
(12, 18)	(1, 0.5, 0.1)	54.1	87.2
(10, 15)	(1, 0.7, 0.3)	54.0	86.3
(10, 15)	(1, 0.3, 0.05)	54.5	87.0

**Table 10:** Sensitivity analysis of scaling coefficient α in Equation (11) on ShanghaiTech PartA dataset. Bold values represent the best results.

α	MAE	MSE
0.2	54.2	86.5
0.25	53.9	86.1
0.3 [Ours]	**53.7**	**85.6**
0.35	54.0	86.2
0.4	54.5	87.0

**Table 11:** Computational complexity comparison on ShanghaiTech PartA dataset. All measurements are conducted under a unified protocol: batch size 1, input resolution 256 × 256, on a single NVIDIA A40 GPU.

Method	Params (M)	FLOPs (G)	FPS
SGANet [18]	21.80	93.07	84.30
CCTrans [24]	107.00	27.32	71.25
CrowdFormer [26]	15.13	23.37	278.4
CrowdUNet [27]	18.4	131.19	68.2
STEERER [37]	64.64	23.51	37.68
Ours	62.8	20.67	30.80

**Table 12:** Cross-dataset generalization results (MAE). Bold values represent the best results.

Method	SHA → QNRF	QNRF → SHA
BL2 [56]	120.3	58.8
STEERER [37]	109.4	54.1
Ours	**99.6**	**54.0**

**Table 13:** Comparative experimental results of multi-level feature fusion selection. Bold values represent the best results.

Scales	MAE	MSE
1/8 scale	61.5	91.6
1/16 scale	54.7	87.2
1/32 scale	55.7	90.4
Layer-wise upsampling	54.8	86.6
Multi-Level Feature Fusion	**53.7**	**85.6**

## Data Availability

The datasets used in this study are publicly available: ShanghaiTech [27], UCF-QNRF [30], UCF_CC_50 [31], and JHU-Crowd++ [32].
